# Dynamic Release from Acetalated Dextran Nanoparticles for Precision Therapy of Inflammation

**DOI:** 10.1021/acsabm.4c00182

**Published:** 2024-05-25

**Authors:** Gizem Erensoy, Loise Råberg, Ula von Mentzer, Luca Dirk Menges, Endri Bardhi, Anna-Karin Hultgård Ekwall, Alexandra Stubelius

**Affiliations:** † Division of Chemical Biology, Department of Life Sciences, Chalmers University of Technology, Gothenburg 412 96, Sweden; ‡ The Rheumatology Clinic, Sahlgrenska University Hospital, Gothenburg 413 45, Sweden; § Department of Rheumatology and Inflammation Research, Institute of Medicine, Sahlgrenska Academy, University of Gothenburg, Gothenburg 413 46, Sweden

**Keywords:** drug delivery, nanoparticles, smart materials, dynamic release, inflammation, rheumatoid arthritis

## Abstract

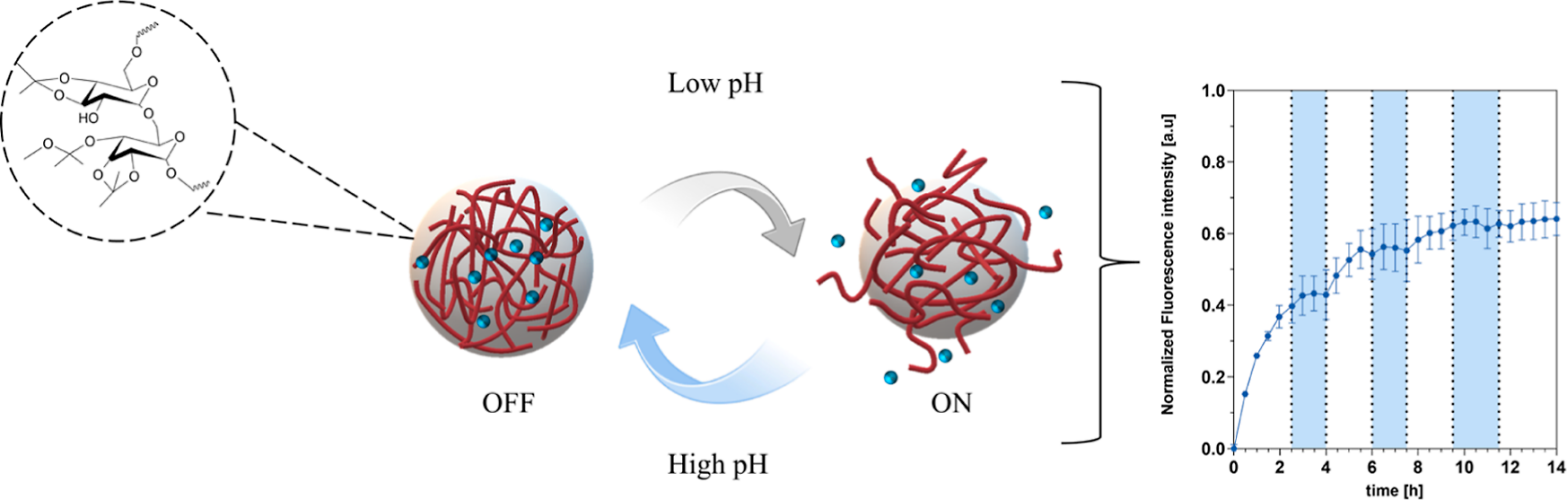

Polymer-based nanoparticles (NPs) that react to altered physiological characteristics have the potential to enhance the delivery of therapeutics to a specific area. These materials can utilize biochemical triggers, such as low pH, which is prone to happen locally in an inflammatory microenvironment due to increased cellular activity. This reduced pH is neutralized when inflammation subsides. For precise delivery of therapeutics to match this dynamic reaction, drug delivery systems (DDS) need to not only release the drug (ON) but also stop the release (OFF) autonomously. In this study, we use a systematic approach to optimize the composition of acetalated dextran (AcDex) NPs to start (ON) and stop (OFF) releasing model cargo, depending on local pH changes. By mixing ratios of AcDex polymers (mixed NPs), we achieved a highly sensitive material that was able to rapidly release cargo when going from pH 7.4 to pH 6.0. At the same time, the mix also offered a stable composition that enabled a rapid ON/OFF/ON/OFF switching within this narrow pH range in only 90 min. These mixed NPs were also sensitive to biological pH changes, with increased release in the presence of inflammatory cells compared to healthy cells. Such precise and controllable characteristics of a DDS position mixed NPs as a potential treatment platform to inhibit disease flare-ups, reducing both systemic and local side effects to offer a superior treatment option for inflammation compared to conventional systems.

## Introduction

Polymeric nanoparticles (NPs) can be designed to achieve advanced and intelligent drug delivery platforms for next-generation therapeutics. By encapsulating and stabilizing compounds such as potent anti-inflammatory glucocorticoids or nonsteroidal anti-inflammatory drugs (NSAIDs), these NPs can achieve precise control of drug release for specific treatments of inflamed tissues.^1^ To date, a variety of systems have been developed including liposomes,^2,3^ dendrimers,^4,5^ and NPs^6−8^ that respond to different biochemical signals. Cells that promote and upregulate inflammation, along with their increased metabolic byproducts, can lead to a shift from a normal physiological pH of 7.4 to a more acidic pH of 6.0.

This shift in pH can be harnessed as an acid-triggered release mechanism when designing NPs as specific drug delivery systems (DDSs). For inflammation, the switch from an ON-state of the DDS to an OFF-state should be rapid due to the rapidly developing inflammatory reaction. To impart specificity, a DDS that can also autonomously be turned OFF would result in an even higher therapeutic efficacy, specifically for diseases with flares such as rheumatoid arthritis (RA). It would minimize unnecessary drug release not only in low disease activity areas but also during low disease activity periods, thereby extending the potential duration of an administered dose. While several examples are achieving the ON state of release from DDS, very few examples can also cease the release from the DDS.^9−11^

For the most common type of inflammatory arthritis, RA, the first treatment strategy is to stop joint inflammation as quickly as possible to prevent or slow down the pace of joint damage.^12^ Despite modern RA therapies, including conventional synthetic, biological, and novel targeted synthetic disease-modifying antirheumatic drugs (DMARDs), having made significant progress toward achieving disease remission without joint deformity, RA remains poorly controlled in up to 30% of patients.^13,14^ To relieve pain and prevent further damage several different potent therapies are used, including glucocorticoids or NSAIDs in addition to DMARDs.^15,16^ However, use of these agents must be balanced to the risk of gastrointestinal bleeding and renal dysfunction, hyperglycemia, increased risk for infection, and other side effects.^17,18^ Additionally, poor absorption, rapid first-pass effects, and high elimination rates limit the use and benefit of these agents.^19^ Investigations into personalized therapy for RA patients have therefore been spurred due to both the patient disease variability and unpredictable treatment response.^20^ An option to achieve a precision treatment strategy could be composed of a DDS that utilizes the disease’s pathogenic mechanisms, such as low pH, against itself.^21^ Specifically, for inflammation in the joint space, the pH typically ranges from 7.4 to 6.0.^22−24^

pH responsiveness can be imparted into DDS with acid cleavable bonds such as polyortho ester, hydrazone, vinyl/silyl ether, boronate, and acetal/ketal groups.^18^ A majority of acetal/ketal compounds do not produce acidic byproducts following hydrolysis thus preventing the occurrence of proinflammatory effects.^25,26^ Acetal/ketal groups can easily be integrated into the structures of dendrimers or polymers like chitosan^27^ and dextran.^28,29^ Acetalated dextran (AcDex) is one of the most investigated dextran derivatives and was first reported by Bachelder and co-workers.^28^ After acetalation of the pendant hydroxyl groups on dextran, the resulting polymer AcDex becomes hydrophobic. The hydrophobicity makes AcDex favorable for drug encapsulation, and a unique feature of AcDex compared to other pH-responsive materials is the highly tunable degradation rate which is dependent on the two different acid labile groups (cyclic and acyclic).^30,31^ Tailoring the cyclic/acyclic ratio is achieved by varying the reaction time, where more thermodynamically stable products are obtained by longer reaction times. These properties make AcDex an ideal system for achieving a highly responsive DDS that can be tuned towards the pH of inflammation, which we and others have shown can be achieved for inflammatory joint diseases.^6,32^ The criteria for an autonomously controlled DDS for inflammation include: (1) rapid ON/OFF kinetics, (2) stability at pH 7.4, and (3) biologically relevant sensitivity and specificity. No system fulfilling these criteria has previously been achieved with pH-responsive DDS. Our study shows that to comply with the specified criteria, a specialized AcDex NP formulation is needed, where a strategy of mixing different pH sensitivities into a mixed NP display superior properties and acts as an autonomously triggered DDS. These controllable characteristics position this system as a potential treatment platform that works against dynamic inflammatory flares.

## Materials and Methods

### Materials

2-Methoxypropene (Acros Organics), acetic acid (deuterated) (Sigma-Aldrich, Merck KGaA, Darmstadt, Germany), acetone (Fisher Scientific, Waltham USA), citric acid monohydrate (Sigma-Aldrich Merck KGaA, Darmstadt, Germany), d-(+)-trehalose dihydrate (Alfa Aesar, Massachusetts, USA), dextran (from *Leuconostoc mesenteroides*, 9–11 kDa) (Sigma-Aldrich, Merck KGaA, Darmstadt, Germany), dichloromethane (Sigma-Aldrich, Merck KGaA, Darmstadt, Germany), 2′,7′-dichlorofluorescein diacetate (DCFH-DA) (Sigma-Aldrich, Merck KGaA, Darmstadt, Germany), dimethyl sulfoxide (deuterated, Sigma-Aldrich, Merck KGaA, Darmstadt, Germany), dexamethasone-fluorescein isothiocyanate (DXM-FITC, Invitrogen, Carlsbad, California), Dulbecco’s modified Eagle’s serum (DMEM, Gibco, Paisley, UK), fetal bovine serum (FBS, Gibco, Paisley, UK), fluorescein isothiocyanate FITC (Sigma-Aldrich, Merck KGaA, Darmstadt, Germany), 1% GlutaMAX (Gibco, Grand Island, USA), IL-1β (GeneTex INC, USA), lipopolysaccharide (LPS, Sigma-Aldrich, Merck KGaA, Darmstadt, Germany), nile red (NR, Sigma-Aldrich, Merck KGaA, Darmstadt, Germany), phosphate buffered saline (PBS w/o Mg2^+^/Ca2^+^, Gibco, Paisley, UK), poly(vinyl alcohol) (PVA, 30,000–70,000 MW, Sigma-Aldrich, Merck KGaA, Darmstadt, Germany), pyridinium *p*-toluenesulfonate (Acros Organics, Antwerp, Belgium), gentamicin and 1% penicillin–streptomycin (Gibco, Paisley UK), resazurin (Alfa Aesar, Massachusetts, USA), sodium hydroxide (Fisher Scientific, Waltham USA), triethylamine (TEA) (Fischer Scientific, Waltham USA), TNF-α (Sigma-Aldrich, Merck KGaA, Darmstadt, Germany), tween 20 (Fisher Bioreagents, Waltham USA).

### Synthesis and Characterization of AcDex Polymers

AcDex polymers were synthesized according to Bachelder and co-workers.^29^ Briefly, dextran (1 g, 0.095 mmol) was dissolved in anhydrous DMSO. Then, pyridinium *p*-toluenesulfonate (15.6 mg, 0.062 mmol) was added to the solution. To start the reaction, 2-methoxypropene (3.4 mL, 37 mmol) was added. The reaction was stopped with TEA (0.5 mL) at various predetermined time points (7, 8, and 30 min for AcDex 35, 58, and 70%, respectively). The product was precipitated with double distilled (dd)-water (pH 9, adjusted with 2% TEA) and isolated by centrifugation and lyophilized for 2 days. The chemical modification of dextran was characterized by ^1^H NMR (Agilent VnmrS, 400 MHz). For NMR analysis, the polymer was suspended in deuterium oxide and acetic acid-*d*_4_ (1:2). The cyclic-to-acyclic acetal ratio was calculated by comparing the peaks of acetone and/or methanol resulting from hydrolysis of AcDex with dextran –OH peaks.

### Synthesis and Characterization of AcDex NPs

NPs were prepared by nanoprecipitation.^33^ Briefly, AcDex polymer was dissolved in acetone (20 mg/mL) and added to a 1% PVA (60 mL) (pH 9, adjusted with 2% TEA) solution by a syringe pump (KDS-100CE, KD Scientific, Holliston, USA). The flow rate was set to 6 mL/h. This mixture was stirred for 2 h to evaporate any residual organic solvent. For mixed NPs, the AcDex 35% and 70% polymers were blended at a 1:1 (w/w) ratio. After 2 h, the NP suspension was diluted and filtered [tangential flow filtration, 500 kDa poly(ether sulfone) membrane] three times with basic dd-water (pH 9). Finally, the dispersion was lyophilized for 2 days to yield AcDex NPs as a fluffy white solid. For loaded NPs the same procedure was used; however, a cargo (NR, DCFH-DA or DXM-FITC) (10% w/w) was added to the organic phase. The loading capacity (LC) and encapsulation efficiency (EE) of NPs were determined by resuspending prewashed NPs (resuspended in dd-H_2_O and centrifuged at 14,000 rpm, 4 °C, 15 min) in acetone followed by incubation for 2 h. The NP suspension was diluted to 1:5, 1:10, or 1:50, and 200 μL of dilutions were transferred to a 96-well plate (polystyrene, flat bottom). The fluorescence intensity of NR was measured at λ_ex_ = 552–20 nm, λ_em_ = 638–50 nm using a microplate reader (Hidex Sense, Oy, Finland). The concentration of the cargo was determined based on the calibration curve of NR obtained from a concentration range of 50–500 ng/mL. To calculate DCFH-DA and DXM-FITC loading, NPs were prepared as above. For DCFH-DA and DXM-FITC, the standard calibration curve was prepared between 50–1000 g/mL and 10–1000 ng/mL, respectively. The fluorescence intensity was measured at λ_ex_ = 490–20, λ_em_ = 535–20 nm for DCFH-DA and λ_ex_ = 485–10 nm, λ_em_ = 535–20 nm and DXM-FITC. The LC and EE were calculated using the following formulations
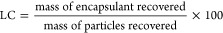

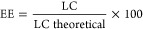


The hydrodynamic size and zeta potential were measured by dynamic light scattering (DLS) (Zetasizer Ultra, Malvern Instruments Ltd., UK). Size measurements were performed in disposable polystyrene UV Micro cuvettes (VWR, Radnor, USA) and measurements of zeta potential were performed using Folded Capillary Zeta Cell DTS1070 (Malvern Instruments Ltd., UK). The NPs were diluted to 0.5 mg/mL in PBS.

The physical stability of the AcDex NPs was evaluated by monitoring the size change over time (up to 96 h). Particles were incubated at 37 °C with constant shaking at 100 rpm in PBS (pH 7.4, 6.5, and 6, adjusted with 0.1 M citric acid). At determined time points (0, 1, 2, 4, 24, 48, 72, and 86 h) the NPs’ size were measured by DLS.

The residual PVA amount of the NPs was calculated according to Spek et al.^34^ Briefly, a 10 mg/mL concentration of NP suspension was prepared in dd-H_2_O. 700 μL of this suspension was hydrolyzed by adding 100 μL HCl (1 M) and incubated at 60 °C for 15 min. Neutralization was done by adding the same amount of NaOH (1 M). After adding 100 μL water for dilution, complex formation was initiated by adding 600 μL boric acid solution (0.65 mol/L) and 100 μL Lugol’s solution (0.016 mol/L potassium iodide and 0.01 mol iodine). Samples were incubated for 15 min to allow a complete complex reaction and then absorbance was measured at 690 nm. Analysis was made based on a standard curve of PVA between 9.375 and 93.75 μg/mL.

Scanning electron microscopy (SEM, Jeol-JSM 7800F, 30 kV, JEOL Ltd., Japan) confirmed the size and morphology of the NPs. Particle samples were prepared by dropping 10 μL of a NP suspension (0.5 mg/mL in water) onto a Mica disc (12 mm, highest grade V1, Ted Pella Inc.) which were attached to stages with double sided carbon tape. The coatings were left to dry under vacuum overnight. A 4 nm gold layer was sputtered onto the dried samples to keep them from being charged by the electron beam of the microscope. Images were analyzed by Fiji ImageJ (NIH, version 2.3.0).

### Release of Cargo from AcDex NPs

NR loaded NPs were dissolved in PBS (pH 7.4, 2.5 mg/mL) and centrifuged (14,000 rpm, 4 °C, 15 min). The pellet was dispersed in the same buffer (1 mL) by vigorous pipetting, vortexing, and ultrasonication for 1–3 min. To ensure that NR could be released into a hydrophilic environment, release was conducted with 150 μL of buffer solution (PBS, 0.01% Tween 20, at pH 7.4 and 6) which was pipetted in triplicates into a 96-well plate. A volume of 50 μL of NPs was mixed with buffer solutions and measurement started immediately (λ_ex_ = 552–20 nm, λ_em_ = 638–50 nm). At intervals of 30 min, a measurement was taken, and this block of time was identified as a cycle. The release experiments were conducted in triplicate.

ON/OFF/ON release was performed by adding a base (0.1 M NaOH) and acid (0.1 M citric acid) at distinct time points during the release studies as mentioned above. The experiment started at the ON-state (pH 6). Measurements were taken at 30 min intervals, defining each period as a cycle. Before cycles 6, 13, and 20, 11.46 μL of base was added to the wells to mimic the OFF-state, (resulting in pH 7.4). To iterate the ON-state (pH 6), before cycles 9, 16, and 24, 5.4 μL of citric acid was added. After cycle 24, measurements were conducted without further interruption. The normalized drug release was calculated as follows
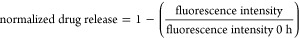


### Drug Release Kinetics

Mathematical models were used to examine, interpret, and compare the release kinetics of a model cargo. Drug release kinetics of NPs were determined by zero-order, first-order, and Korsmeyer–Peppas mathematical models, respectively, as follows
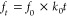

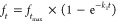

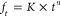


The fractional amount of model cargo released at time *t* is represented by *f*_*t*_. In the equations, the representations of *K*_0_, *K*_1,_ and *K* are the kinetic constants of the zero-order, first-order, Korsmeyer–Peppas. “*n*”, the diffusion exponent, is indicative of the drug release mechanism. The collected cumulative release data were fitted to the above-mentioned models using MATLAB R2022b software (MathWorks Inc.).

### In Vitro Release

For in vitro release studies, RAW 264.7 macrophages (Sigma-Aldrich) were cultured in DMEM supplemented with 10% FBS and 1% penicillin–streptomycin. The cells were maintained under a humidified atmosphere at 37 °C with 5% CO_2_. The cells were grown in 25 cm^2^ tissue culture flasks (TPP, Switzerland) and subcultured every 2–3 days. For the release assay, cells were seeded between passages 7–10 and adhered overnight at a density of 5 × 10^4^ cell per well in a 48-well plate (VWR, USA). 1 μg/mL LPS in media was added to designated wells, and media was used as control. The cells were prestimulated for 4 h. DCFH-DA was dissolved at a concentration of 5 mM in DMSO, then diluted to a concentration of 5 μM. The DCFH-DA-loaded AcDex 70% and mixed NPs, were washed three times with 2 mL of PBS and centrifuged for 10 min, at 14,000 rpm, 4 °C to remove free DCFH-DA. NPs were diluted to 1 mg/mL. Before addition of NPs, the wells were washed three times with PBS to remove the culture medium. Then, 80 μL PBS ± LPS, 20 μL resazurin (0.15 mg/mL), and 100 μL DCFH-DA or DCFH-DA loaded NPs were added to designated wells for a final volume of 200 μL. To reduce interference by the culture media and FBS all experiments were conducted in PBS for 4 h due to the lack of nutrients.^35^ The final concentration in the wells was 2.5 μM DCFH-DA, 0.5 mg/mL for the NPs, and 1 μg/mL for LPS. The fluorescence measurements were started immediately in a microplate reader (Hidex) on a kinetic program at 37 °C, measuring every 10 min for 4 h. The excitation wavelength (λ_ex_) for DCF was set to 490 ± 20 nm and an emission wavelength (λ_em_) of 544 ± 20 nm. Fluorescence intensity was calculated by subtracting the mean background intensity at each time point to account for the spontaneous conversion of DCFH-DA to DCF. Conversion to DCF by control and LPS-stimulated cells was compared by normalizing the area under the curve (AUC) to the control cells.

### Patient-Derived Cell Studies

Primary fibroblast-like synoviocytes (FLS) were isolated from synovial tissue specimens from a patient with inflammatory arthritis undergoing joint replacement surgery (Sahlgrenska University Hospital, Gothenburg, Sweden, Ethical approval Dnr: 573–07). The cells were cultured in DMEM with GlutaMAX, 10% heat-inactivated FBS, 50 mg/mL Gentamicin, and 100 U/ml penicillin–streptomycin. Cells were seeded 5 × 10^4^ cells per well in a 96-well plate and stimulated with IL-1β + TNF-α (5 ng/mL) followed by the addition of the treatments of free drug DXM FITC, and DXM-FITC loaded mixed NPs, and AcDex 70% NPs for another 24 h. Then, resazurin (0.15 mg/mL) was added and incubated for another 3 h. Cell viability was analyzed by detecting resorufin (λ_ex_ = 540 ± 20 nm, λ_em_ = 590 ± 20 nm) using a micro plate reader (Hidex). Cell viability is expressed as a percentage of unstimulated cells.

### Statistical Analysis

Statistical analysis was performed for the in vitro release by comparing the AUC between unstimulated cells and LPS-stimulated cells using an unpaired *t*-test. Cell viability data was compared by ordinary two-way analysis of variance (ANOVA) complimented with Tukey’s test using GraphPad Prism [version 10.1.0 (264)].

## Results and Discussion

### Synthesis of AcDex Polymers and NPs

The chemical modification of dextran is fundamental for controlling the pH-dependent degradation and release properties. Increasingly hydrophobic AcDex polymers were synthesized by varying reaction times for 7, 8 and 30 min.^29^ The acetal groups are prone to hydrolysis which regenerates native dextran and innocuous amount of acetone and methanol as small molecule byproducts.^29^^1^H NMR data confirmed that the polymers were modified with acyclic and cyclic acetal groups with sharp acetone (2.2 ppm) and methanol peaks (3.37 ppm; Figures S1–S3). By comparing these peaks with the dextran–OH peaks (between 3.4 and 4 ppm) the acetalation degree of AcDex polymers was calculated as 35, 58, and 70% respectively. The most important parameter that influences the degradation rate is the percentage of cyclic groups on the polymer since they degrade slower than the acyclic ones, and provide the rate limited release mechanism.^29^ To understand the degradation profile of the different polymers, they were incubated at different pH values in PBS (7.4, 6.5, and 6) and the amount of released dextran was measured by the bicinchoninic acid assay (BCA) assay (Figure S4). As expected, degradation of AcDex 35, 58, and 70% polymers increased in more acidic environments. AcDex 70% polymer showed high stability and low reactivity due to the higher number of cyclic groups, making it a slower responding DDS. AcDex 35% and AcDex 58% demonstrated pH dependent reactivity after 4 h whereas this period has been 24 h for AcDex 70%. These results support that polymers that had been synthesized over a longer period were degraded at a significantly slower pace.

**Figure 1:**
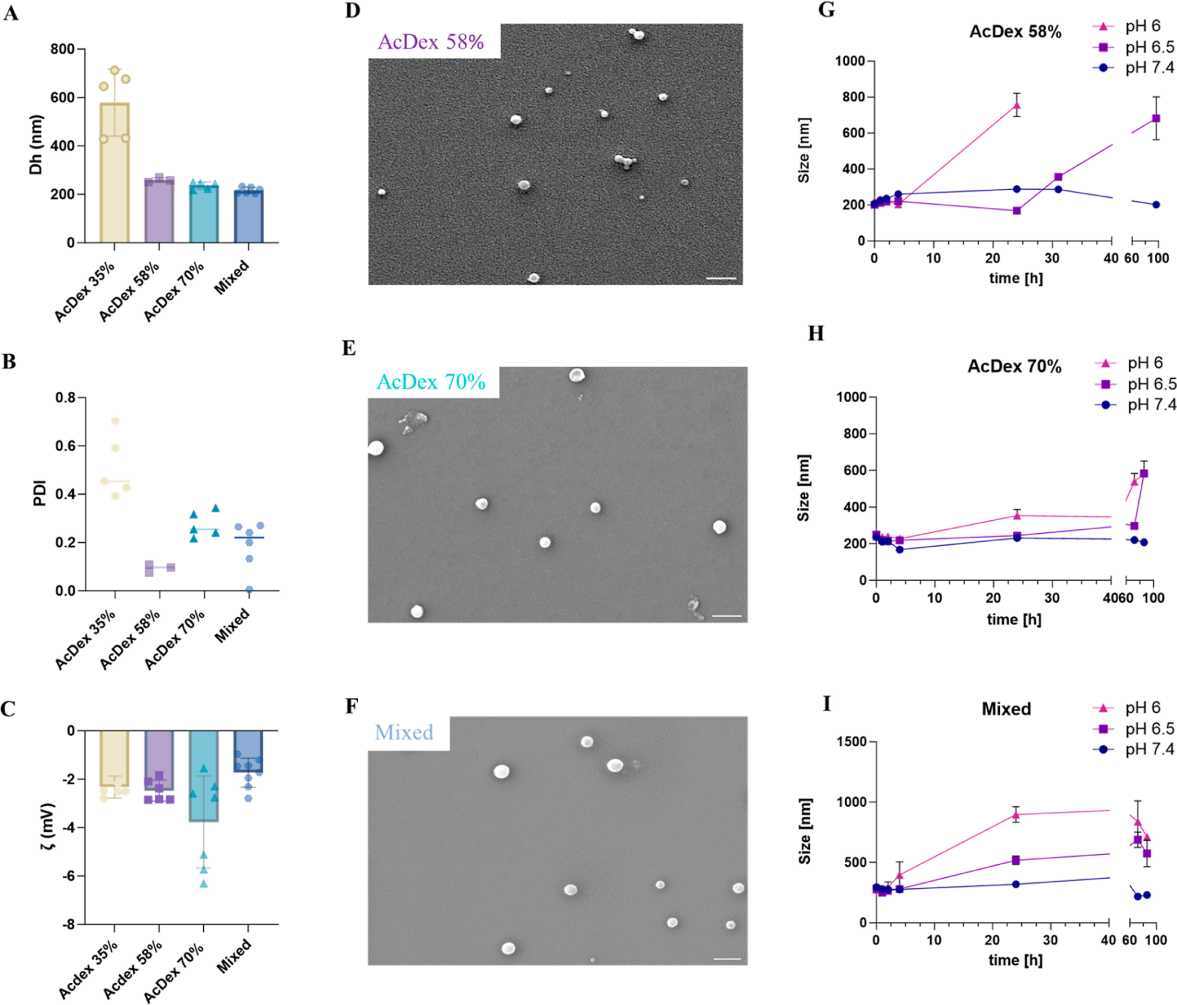
Characterization of AcDex NPs. (A) The hydrodynamic diameter, (B) polydispersity index (PDI), and (C) zeta potentials of AcDex 35, 58, 70%, and mixed NPs were quantified in PBS using DLS. SEM analysis of (D) AcDex 58%, (E) AcDex 70%, and (F) mixed NPs sizes, scale bar set to 500 nm. Hydrodynamic diameter size data for (G) AcDex 58%, (H) AcDex 70%, and (I) mixed NPs over 96 h in PBS at different pH values. The data are presented as mean ± S.D., (*n* = 3).

All AcDex NPs were synthesized by nanoprecipitation. Three different PVA concentrations (0, 0.3, and 1%) were evaluated in the formulation optimization process as NPs prepared without PVA resulted in large polydispersity indexes (PDI values ranging between 0.422 and 0.767) indicating an unstable formulation. While including PVA as a surfactant slightly increased NP sizes,^36,37^ 1% PVA inclusion still produced appropriate NPs sizes and PDI and was necessary to prevent aggregation by stabilizing the dispersion. The final residual amount of PVA was quantified to 29.7 and 33.2 μg/mg NP for AcDex 70% and mixed NPs, respectively, equaling final PVA concentrations of 2.97 and 3.32%. All formulations prepared resulted in monodispersed particles based on their hydrodynamic size (*D*_h_), ranging from 200 to 300 nm and narrow PDI values except AcDex 35% (Figure 1A,B). Due to the AcDex 35% constituting NPs polydispersity, the AcDex 35% polymer was deemed too hydrophilic to form NPs by itself and was excluded from further studies. Instead, AcDex 35% polymer was included as a composite NP in combination with AcDex 70% at a 1:1 mix (mixed NPs) designed to achieve a release rate that is neither fast nor excessively slow. The 1:1 mix was the minimal ratio that allowed for a stable formation of NPs, as a ratio of 1.5:0.5 (AcDex 35%: AcDex 70%) demonstrated similar instabilities as those of the pure AcDex 35% NPs (data not shown).

The zeta potentials of the NPs were in the negative range (−4 and −1.83 mV) and could be considered neutral (Figure 1C). The usage of high PVA surfactant during the formulation step may lead to neutral zeta potential since it interacts with the polymer chains and affects the surface potential.^38,39^ Sizes of all particles were confirmed by SEM (Figure 1D–F). SEM investigations revealed that these NPs are homogeneous and spherical. The mean size of NPs was calculated as 143.9 ± 48, 191.4 ± 45, and 214.5 ± 49 nm for AcDex 58%, AcDex 70% and mixed NPs, respectively.

Stability of the NPs was evaluated at pH 7.4 (physiological pH), 6.5, and 6 (using citric acid) over 96 h since pH plays an important role in release responsiveness. To demonstrate the feasibility of the NPs for arthritic diseases, the specificity and sensitivity of the NPs were assayed in the pH range of 6.0–7.4, which aligns with the acidity levels observed in the joint environment.^22−24^ AcDex 58% NPs were stable at pH 7.4 and started to degrade after 30 h at pH 6.5, and already after 24 h at pH 6 (Figure 1G). As expected, AcDex 70% NPs displayed high stability at pH 7.4 and started to degrade after 48 h at pH 6, and after 86 h at pH 6.5 (Figure 1H). Only slight variations in size were observed for these NPs at pH 7.4, indicating that the NPs have high stability under physiological conditions, which was also true for mixed NPs (Figure 1I). The findings also indicated the successful formulation of NPs in terms of pH responsiveness, as the NPs changed in size at lower pH. Compared to both the 70 and the 58% NPs, the size of mixed NPs started to increase already within 4 h at pH 6 and after 24 h at pH 6.5, indicating a more sensitive pH-dependent degradation profile. It could be observed that the NPs size decreased slightly within the first 4 h before starting to swell and increase in size. In an acidic environment the rate of this process was even faster. This process is consistent with a surface erosion mechanism,^40,41^ where the surface polymers first react to convert into water-soluble dextran and undergo a subsequent layer-wise degradation. However, disassembly, swelling, and degradation have also been suggested as mechanisms for responsive polymeric matrices.^42^ DLS measurements after 24 h showed a higher polydispersity and different size populations, confirming the swelling and degradation of the NPs.

### pH-Responsive Cargo Release

To demonstrate the ability of AcDex NPs to encapsulate and release a model cargo, NR was loaded into the NPs. NR is a hydrophobic dye that is fluorescent in hydrophobic environments but quenched in aqueous solution.^43^ The emission of NR-loaded AcDex NPs and free NR was monitored, and NPs showed higher intensity over the spectrum than free NR (Figure S5). The LC of NR was calculated to 1.67, 1.94, and 1.19% for AcDex 58, 70%, and mixed NPs, respectively, matching LC to the hydrophobicity of the polymers of the NPs, as expected.

**Figure 2:**
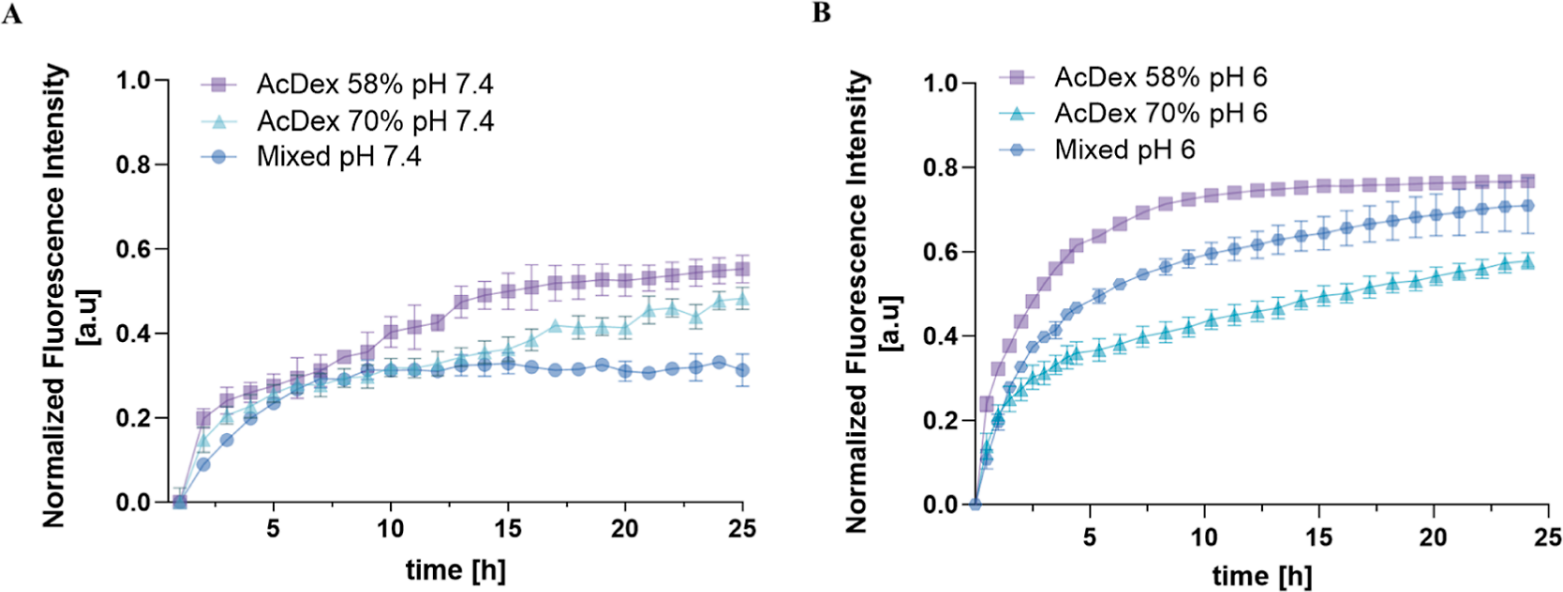
Release profile for NPs. (A) Comparison between AcDex 58%, AcDex 70%, and mixed NPs at pH 7.4. (B) at pH 6 PBS. Fluorescence intensity of NR was monitored up to 24 h. The data are presented as mean ± SD for AcDex 58% (*n* = 1) and AcDex 70% and mixed NPs (*n* = 3).

AcDex 58% NPs demonstrated NR release at pH 7.4 up to 55% (Figure 2A), consistent with previous findings.^44,45^ The release could result from NR being adsorbed to the surface and, thus, being released without control. The release of hydrophobic molecules from a carrier material that contains PVA can be influenced by several factors, such as drug carrier interactions, size, and shape of model cargo. PVA is a hydrophilic polymer, and model cargos like NR can form weak and reversible binding interactions including van der Waals, hydrophobic interactions with PVA.^46^ During the initial phase of release, the model cargo present at or near the surface of the NP can be easily released due to these weak interactions, which could explain the initial burst release. Alternatively, the initial burst release could be due to a swelling process, which was also indicated by the stability measurements. Nevertheless, the data suggest the need for a more stable material than NPs consisting of polymer AcDex 58%. Such a spontaneous release was also noted with the AcDex 70% NPs, despite AcDex 70% being more hydrophobic than AcDex 58%. However, NR release from the mixed NPs was significantly lower and only reached 30% at pH 7.4 (Figure 2A).

We further compared the release profiles at pH 6 (Figure 2B). About 45% of the model drug was released from AcDex 70% NPs, whereas during the same period about 60% of the model cargo was released from mixed NPs, indicating that mixed NPs are more sensitive to pH. Despite AcDex 58% demonstrated a better release profile at pH 6 than AcDex 70% and mixed NPs, considering the spontaneous release observed at pH 7.4 for AcDex 58% (up to 55% at 24 h), mixed NPs demonstrated an enhanced overall release and stability profile. The altered release behavior of the mixed NPs compared to pure AcDex 70% NPs suggests a combination of the two polymers in the final NP composition, as indicated in Figure 1H–I. This implies an even mix of the polymers within the NPs. If the AcDex 35% polymer was only adsorbed to the surface, we would expect a two-step release profile; an initial faster release rate followed by stabilization into a slower release rate. However, the mixed NPs display a continuous release rate over time that is faster than that of the pure AcDex 70% NPs.

**Figure 3:**
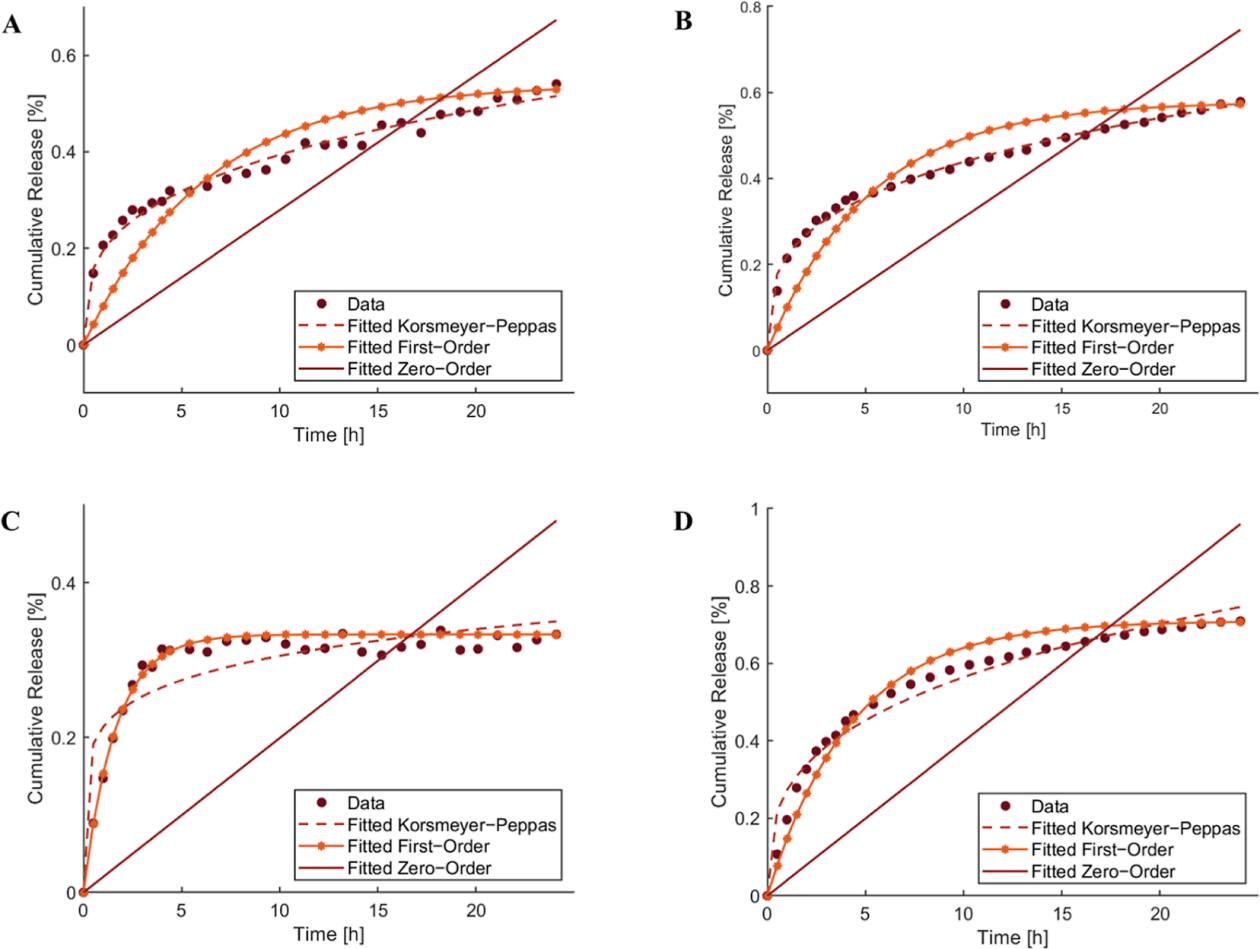
Zero-order, first-order, and Korsmeyer–Peppas kinetic models calculated for AcDex 70% at (A) pH 7.4, (B) pH 6 and for mixed NPs at (C) pH 7.4, (D) pH 6.

As a result, we decided to proceed with mixed NPs, designating AcDex 70% NPs as the control material due to its hydrophobicity compared to mixed NPs, and representing an internal control. While AcDex 70% NPs displayed greater stability across various pH levels, we were able to compare the behaviors of two similar NPs that differed only in one constituent polymer, strengthening our findings and conclusions.

To determine a potential release mechanism for AcDex 70% and mixed NPs, the release data was evaluated using different reaction kinetics comparing zero-order, first-order, or Korsmeyer–Peppas models (Figure 3). The correlation coefficient (*R*^2^) values were determined using the equations of kinetic models and are summarized in Table 1. Zero-order release kinetics refer to the process of constant release independent of concentration, whereas the first-order model states that the change in the concentration with respect to change on time relies solely on the concentration itself. The Korsmeyer–Peppas model was developed to specifically model the release of model cargo from a polymeric matrix. It can be used to interpret release mechanisms such as diffusion controlled and degradation-controlled release. This model allows for simultaneous consideration of the diffusion of water into the device and cargo out of the system.^47,48^ The model involves two parameters; *K*, which incorporates structural modifications and geometrical characteristics of the system, and *n* which is the exponent of release and is related to the drug release mechanism. A value of *n* < 0.45 indicates Fickian diffusion and a nonswellable matrix-diffusion; 0.45 < *n* < 1.0 indicates anomalous (non-Fickian) transport, such as both diffusion and erosion; and *n* = 1 characterizes zero-order release behavior.

**Table 1:** Correlation Coefficient *R*^2^ of NR Release from NPs for Different Release Kinetics Models

	regression coefficient *R*^2^ value							
	zero-order		first-order		Korsmeyer–Peppas		diffusion exponent *n*	
nanoparticle	pH 6	pH 7.4	pH 6	pH 7.4	pH 6	pH 7.4	pH 6	pH 7.4
AcDex 70%	–0.1998	–0.1486	0.8299	0.7557	0.9941	0.9867	0.3022	0.3058
mixed	0.3655	–2.4307	0.9926	0.9751	0.9721	0.8128	0.4199	0.1554

As expected, the zero-order model did not fit with any of the release profiles at the given pH, since the release kinetics does not follow a linear release-time relationship. The best-fitted model to describe release from mixed NPs was the first-order model and indicated a diffusion-controlled release.^49^ The best-fit model for AcDex 70% was Korsmeyer–Peppas for both pH values enabling a calculation of the diffusion exponent (*n*) value for the initial 60% of the release pattern. Therefore, it is worth mentioning that Korsmeyer–Peppas model is not suitable for describing the entire release profile.^47,50^ The *n* values were determined to be smaller than 0.45 (Table 1) suggesting Fickian diffusion at both pH values for AcDex 70% NPs suggesting possible diffusion and degradation mechanism.^49^ The Fickian diffusion model usually occurs in polymeric matrices with a glass transition temperature (*T*_g_) exceeding the ambient temperature like dextran.^51^ These results correlate well with an initial release of weakly bound dye followed by more constant release kinetics influenced by the diffusion and degradation of AcDex 70% and mixed NPs.

### Dynamic Release of NPs

**Figure 4:**
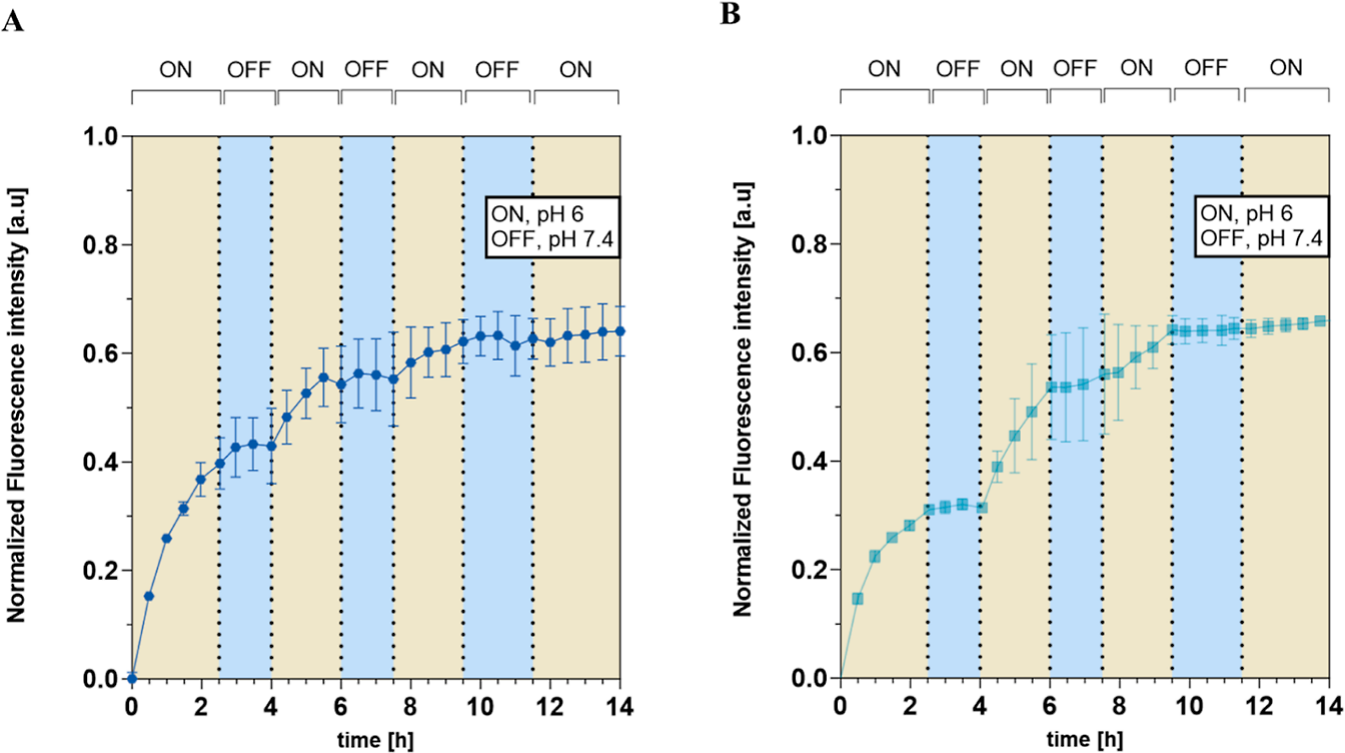
ON/OFF/ON/OFF/ON release for (A) mixed NPs and (B) AcDex 70% NPs. Measurements started at pH 6 (ON state, marked yellow). To induce the OFF-state, NaOH (0.1 M) was added to shift pH to pH 7.4, and measurement continued (marked blue). One M citric acid (1 M) was added to the same wells to shift the pH back to 6 to induce the ON state. The data are represented as mean values ± SD (*n* = 3).

RA and other inflammatory diseases display dynamic disease activity, with flares and periods of low activity. A flare-responsive drug release system could therefore be more beneficial than a sustained release system, as it would only release drugs when the inflammation is ongoing to avoid treating the tissue when it is not needed. An optimized system would be able to remain stable in neutral pH, be rapidly turned ON, and crucially also be turned OFF to stop the release. To mimic this process, we set up a system where the release of the NR from NPs was started at pH 6 (ON-state) and monitored for 2.5 h before shifting pH to 7.4 with 0.1 M NaOH to induce the OFF-state (Figure 4). The OFF-state was monitored for 90 min, and then the environment was acidified with citric acid to trigger the release again. This ON–OFF pH-shifting cycle was repeated two more times. A rapid increase in the fluorescence intensity was detected in the first ON-state. mixed NPs showed faster kinetics (Table 2, release rate = 0.1345 au/h) and higher intensity than AcDex 70% (release rate = 0.1131 au/h) After raising the pH to 7.4, a release stagnation was observed for both NPs, resulting in the same slow release rate for both NPs (Table 2). By decreasing the pH to 6 again, the release rate increased for both NPs, confirming the autonomous release capacity of the NPs. This rapid shift was observed for all further cycles of pH changes, albeit with decreasing release rates over time. Interestingly, despite AcDex 70% NPs demonstrating a slower release profile compared to the mixed NPs in constant reduced pH, they displayed a faster release kinetic when the pH was adjusted between 7.4 and 6.0. The reason could be attributed to residual amounts of PVA, leading to a higher stability and thus slower release rate. Since mixed NPs had a higher amount of residual PVA this may slow down the release compared to AcDex 70% NPs. Another reason might be the higher LC of 70% AcDex 70% NPs. The demonstrated rapid and dynamic changes with high sensitivities toward different pHs are significant for these systems, and allow for highly specific and controllable DDS, able to both turn ON the release, as well as to turn it OFF. While dynamic release from NPs has been shown before,^52^ to the best of our knowledge, this is the first time it has been shown for medical relevant applications.

**Table 2:** Release Rate (au/h) of ON- and OFF-States

	ON	OFF	ON	OFF	ON	OFF	ON
mixed	0.1345	0.0057	0.0696	0.0054	0.0296	–0.0045	0.012
AcDex 70%	0.1131	0.0057	0.0938	0.0055	0.0506	0.0006	0.0056

Particle size plays a crucial role in tuning the rate of drug release. Larger particles have a higher likelihood of encapsulating more therapeutics; however, they can lead to a slower release profile. A benefit of using small NPs compared to bigger particles therefore lies in the possibility of rapid and triggered release due to the higher surface-to-volume ratio, facilitating the quick release of cargo. The rapid release stems both from quick release of cargo encapsulated near or at the surface as well as the rapid diffusion of the cargo trapped in the core. This is facilitated by shorter diffusion distances of the cargo moving outward, and the surrounding media moving inward.^53^ However, in theory, larger particles should have the potential to achieve a greater number of ON/OFF/ON cycles, a possibility that warrants exploration in future studies. In conditions requiring rapid, dynamic release, such as inflammation, achieving a faster release response with smaller NPs is paramount, ensuring a timely and responsive release mechanism tailored to the demands of the inflammatory condition.

### In Vitro Release of AcDex NPs

**Figure 5:**
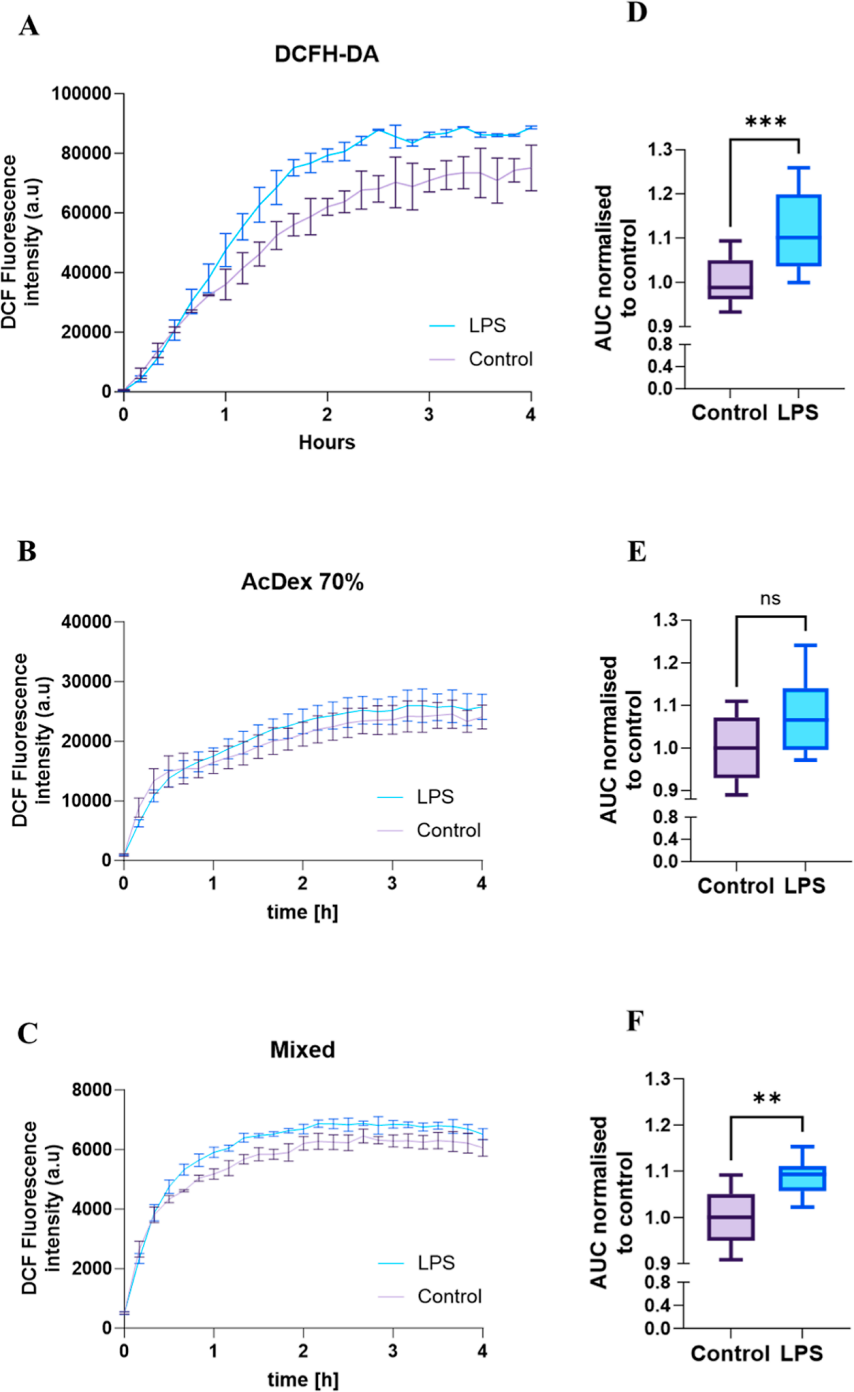
Metabolic conversion of DCFH-DA to DCF by cells was recorded for 4 h. Kinetic measurement of (A) free DCFH-DA, (B) AcDex 70% and (C) mixed NPs. AUC from LPS-stimulated cells was normalized to the control cells (D) DCFH-DA (E) AcDex 70% and (F) mixed NPs. The data are presented as mean ± SD for AcDex 70% and mixed NPs, (*n* = 8), and DCFH-DA (*n* = 12). Unpaired *t*-test was used to analyze the data where **p < 0.01, ***p < 0.001.

To ensure biological biocompatibility, cytotoxicity studies were performed (Figure S6). We evaluated the in vitro release mechanism of NPs by using macrophages, as they are part of the first line of defense in innate immune system.^54^ To monitor our pH-responsive materials, NPs were loaded with DCFH-DA that is intracellularly converted to the highly fluorescent DCF.^55^ Cells were prestimulated for 4 h with LPS to mimic inflammation and washed, and then the conversion of DCF was monitored and compared. Cell viability was constantly monitored to ensure that the DCF conversion was due to active cell metabolism and not an artifact (Figure S7). As shown in Figure 5A,D, free dye DCF formation was 11.6% higher in LPS-stimulated cells compared to control cells. This outcome indicated that macrophages converted DCFH-DA more efficiently into DCF under inflammatory conditions.

Cells treated with AcDex 70% NPs demonstrated no difference between stimulated and control cells, indicating that these NPs are too hydrophobic and not reactive enough to achieve sensitivity toward the rapid inflammatory environment (Figure 5B,E). Mixed NPs showed a higher DCF formation, meaning that the disease activity led to a higher release from the NPs, and thereby a higher conversion of DCFH-DA to DCF was observed (Figure 5C). In addition, the mixed NPs produced a fast response and a statistically significant difference was detected between the stimulated and control cells (Figure 5F). It is worth mentioning that a slower increase in DCF was observed for the cells treated with NPs compared to free dye, as the dye both needed to be released and converted to produce a signal.

**Figure 6:**
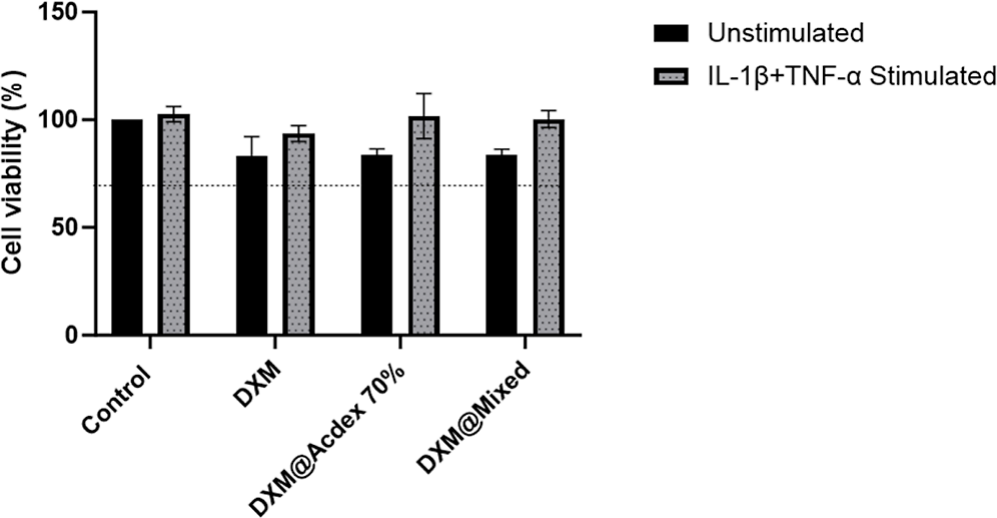
FLS cells were treated for 24 h with either free DXM or DXM encapsulated in NPs. Resazurin was used to measure cell viability after 3 h represents mean ± SD (*n* = 3). The dashed line represents the 70% cytotoxicity limit according to FDA.

In long-term chronic inflammation, the behavior of local immune cells is influenced by the tissue microenvironment. In RA, the FLS are activated and undergo a change from harmless cells to destructive and aggressive cells.^56^ These transformed cells play an important role in the production and progression of RA where they can contribute to joint inflammation and create damage by producing pro-inflammatory cytokines and enzymes that degrade cartilage and bone after stimulation.^57^ To ensure that FLS would not react to the NPs as foreign material or induce potential toxicity, we investigated the tolerability toward the NPs after loading them with DXM.^58,59^ FLS cells were treated with either DXM or DXM@AcDex 70% and DXM@Mixed NPs with or without stimulation (Figure 6). Unstimulated cells were more sensitive than stimulated cells, yet none of the particles induced cytotoxicity under either of the conditions and could be considered safe.

## Conclusions

In summary, we tuned the pH responsive sensitivity of AcDex NPs for drug delivery with the intention to prevent and reduce an inflammatory reaction as close to the onset of a potentially damaging acute inflammatory flare as possible. Since an inflammatory flare occurs rapidly, fast release kinetics to inhibit or reduce inflammation is crucial for minimizing pain and symptoms. The introduction of ON/OFF/ON stimuli responsiveness into the system allowed for a controlled and rapid release. We further demonstrated that the release is favored in an inflamed environment when a specific composite of mixed NPs was formulated. This blend of two polymers enabled both stability and sensitivity that surpassed the NPs composed of a single polymer species, which was also shown to be crucial for achieving a biologically compatible material. As a proof of concept, we investigated the therapeutic potential of AcDex NPs, aiming to decrease inflammatory signaling.

While this platform was evaluated in inflammation focusing on RA, the concept is widely applicable and holds potential for patients suffering from flares in other chronic inflammatory diseases. This involves exposing the drug only in actively inflamed areas, thereby reducing both the usage of high-level drug doses and side effects.

## Supporting Information Available

Characterization of AcDex 35, AcDex 58, and AcDex 70% polymers including ^1^H NMR and BCA characterization; characterization of fluorescence intensity of NR in different environments; cell viability assays (PDF)
