# Supplementary material for: Dynamic Release from Acetalated Dextran Nanoparticles for Precision Therapy of Inflammation
Source: ACS Appl Bio Mater. 2024 May 25;7(6):3810–20. doi: 10.1021/acsabm.4c00182 (PMC11191005; doi:10.1021/acsabm.4c00182)
Supplement: Supplementary file 1 — mt4c00182_si_001.pdf [file mt4c00182_si_001.pdf]

Supporting Information

Dynamic Release from Acetalated Dextran Nanoparticles for Precision Therapy of Inflammation

Gizem Erensoy[a]*, Loise Råberg*[a]*, Ula von Mentzer*[a]*, Luca D. Menges*[a]*, Endri Bardhi*[a]*, Anna-Karin Hultgård Ekwall*[b,c]*, Alexandra Stubelius*[a]***

[a]Department of Life Sciences, Chalmers University of Technology, Gothenburg, Sweden

[b]The Rheumatology Clinic, Sahlgrenska University Hospital, Gothenburg, Sweden

[c]Department of Rheumatology and Inflammation Research, Institute of Medicine, Sahlgrenska Academy, University of Gothenburg, Gothenburg, Sweden

*****Corresponding Author: Alexandra Stubelius, Email: alexandra.stubelius@chalmer.se

Index:

**Figure S1-S3**	^{1}H-NMR spectrums of AcDex polymers	Page 2-3
**Figure S4**	Degradation of AcDex polymers	Page 4
**Figure S5**	Fluorescence intensity of AcDex 58% polymer	Page 5
**Figure S6**	Cell viability of AcDex NPs	Page 6
**Figure S7**	Cell viability of stimulated and control cells	Page 7

1) ^{1}H -NMR spectrums for AcDex polymers

The chemical modification of dextran was characterized by 1H-NMR (Agilent VnmrS,400 MHz). For NMR analysis, the polymer was suspended in deuterium oxide and acetic acid-$_{d4}$(1:2). The cyclic-to-acyclic acetal ratio was calculated by comparing the peaks of acetone and/or methanol with dextran -OH peaks.

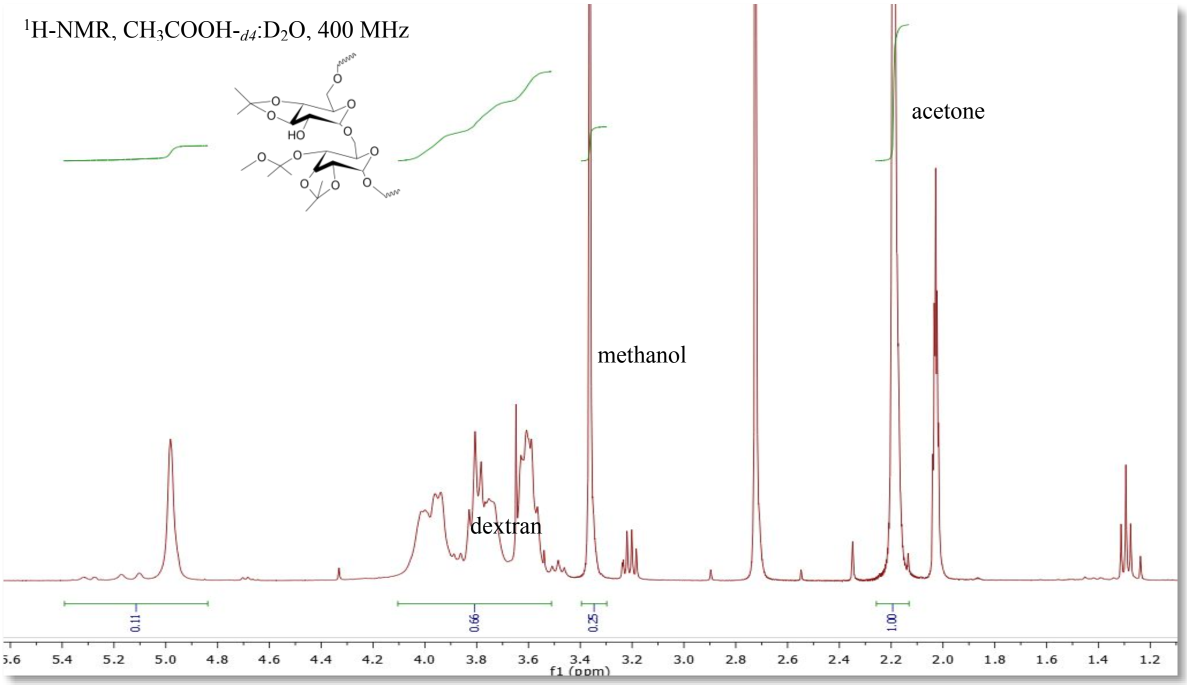

Figure S1. ^{1}H NMR spectrum of AcDex 35% polymer

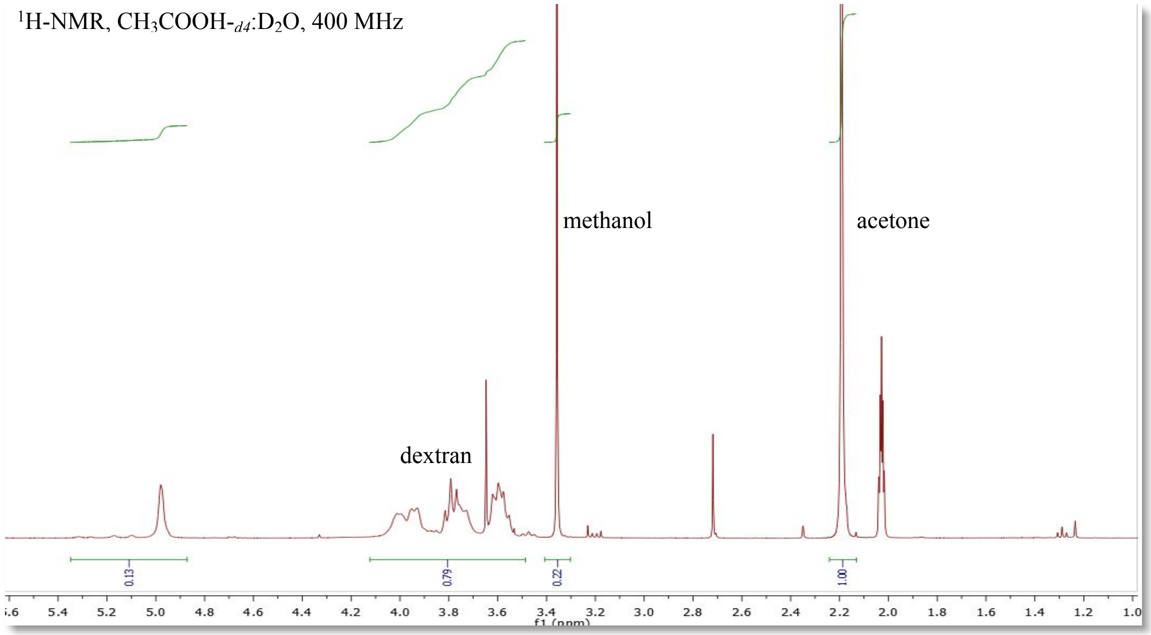

Figure S2. ^{1}H NMR spectrum of AcDex 58% polymer.

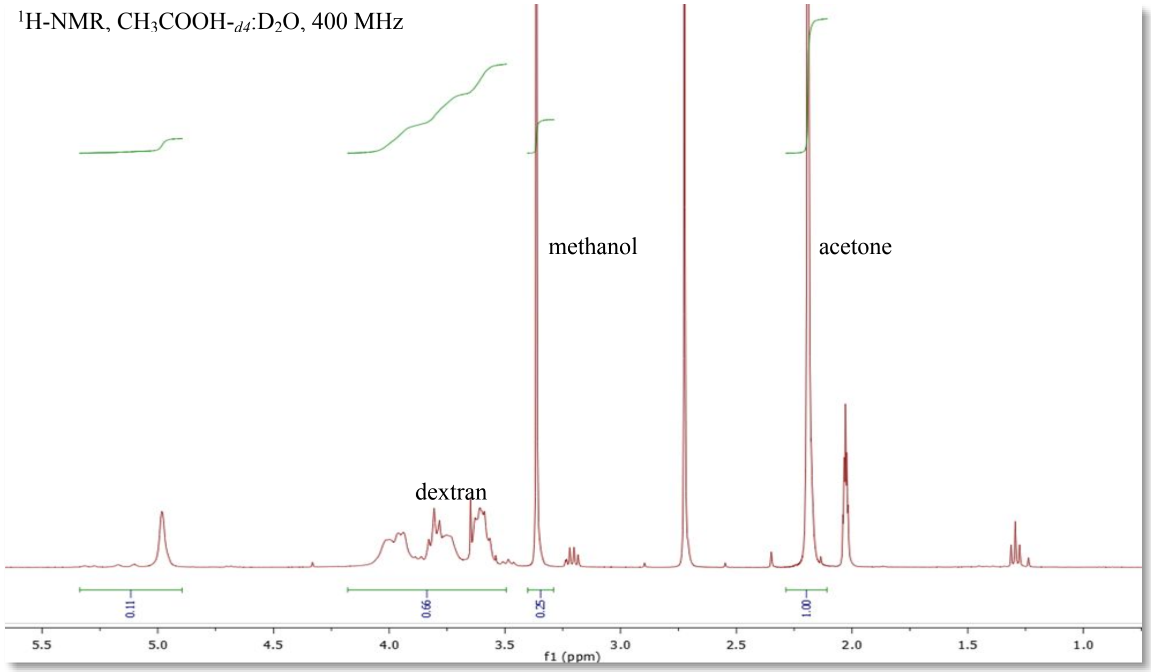

Figure S3. ^{1}H NMR spectrum of AcDex 70% polymer.

2) Polymer degradation

The degradation profile of polymers was tested by bichinchoninic acid copper reduction (BCA) assay. Polymers were suspended in acetone (100 mg/mL), then diluted with PBS (pH 7.4, 6.5, and 6) to a final concentration of 10 mg/mL. The samples were kept at 37 °C and 100 rpm shaking. At various time points samples were centrifugated and aliquots (25µL) of the supernatant were withdrawn and stored at -20 °C in a 96-well plate. After the last time point, samples were thawed and 200 µL working solution was added according to the manufacturer's protocol (Pierce BCA Protein Assay Kit,). Samples were incubated at room temperature for 2 hours and absorbance was measured at 562 nm with a Hidex Sense microplate plate reader (Hidex Oy, Finland).

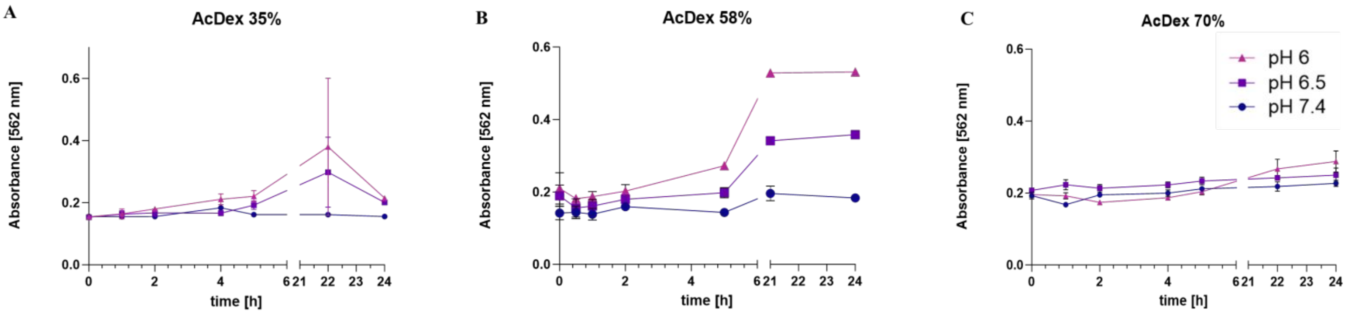

Figure S4. Degradation of A) AcDex 35%, B) AcDex 58% and C) AcDex70% polymers in PBS at different pH. Data are represented as mean values±S.D. (n=3).

Characterization of Fluorescence Intensity of Nile Red in hydrophilic and hydrophobic environments

The emission of Nile red loaded AcDex58% NPs and free Nile red was monitored in a spectral range of 595 nm to 670 nm. Nile Red loaded NPs showed higher FI over the Nile red dispersed in PBS (pH7.4, containing Tween 20 (0.01%)

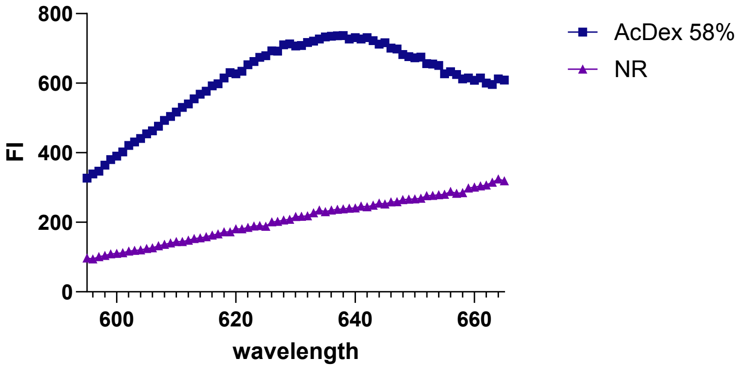

Figure S5. Fluorescence intensity of AcDex58% NPs and Nile Red (free drug).

Cell viability

RAW 264.7 macrophages (Sigma Aldrich) were cultured in DMEM supplemented with 10% FBS and 1% penicillin-streptomycin. The cells were maintained under humidified atmosphere at 37 ℃ with 5% CO_2. Cells were seeded (2×10^4 cells per well, 160 µL) into 96-well cell culture plate (WVR, surface treated, sterile) and let to adhere for 24 hours and treated with 20 µL of NP solutions (dispersed in PBS, 1x, pH 7.4). After 24 hours, resazurin (0.15 mg/mL, 20 µL) was added to each well and incubated for another 3 hours. Cell viability was analyzed by detecting resorufin (λ_{ex}= 540-20 nm, λ_{em}= 590-10 nm) using a micro plate reader (Hidex Sense). Cell viability is expressed as a percentage of media-treated cells.

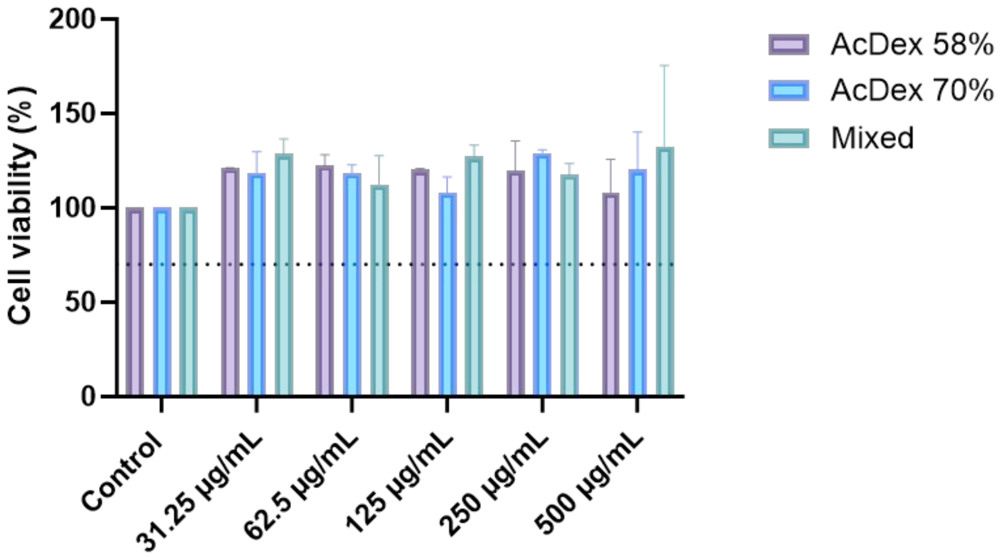

Figure S6. Cell viability of AcDex 58%, AcDex 70% and Mixed NPs was evaluated using the resazurin assay. Cells were seeded in a 96-well plate and treated with NPs. After 24 hours the viability was measured. This experiment was performed three times (n=3) with three technical replicates each time. The dash line represented the 70% cytotoxicity limit. The error bars represent standard deviation of values.

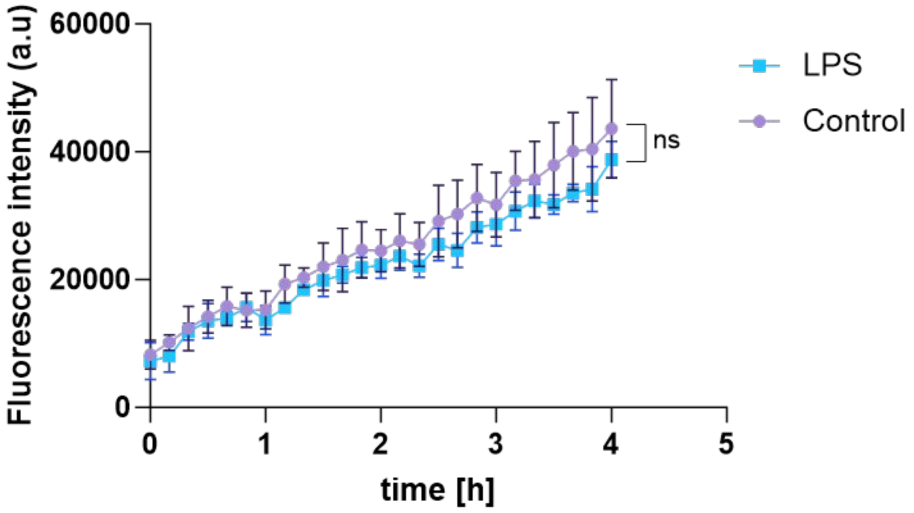

Figure S7. Cell viability of LPS-stimulated and control cells were detected by monitoring resazurin signal during the *in vitro* release experiment Data are represented as mean values±S.D. (n=3). Statistical significance was assessed using an unpaired t-test.
